# Supplementary material for: Pulse-driven self-reconfigurable meta-antennas
Source: Nat Commun. 2023 Feb 6;14:633. doi: 10.1038/s41467-023-36342-1 (PMC9902461; doi:10.1038/s41467-023-36342-1)
Supplement: Supplementary file 1 — Supplementary Information [file 41467_2023_36342_MOESM1_ESM.pdf]

# Supplementary Information

**Pulse-Driven Self-Reconfigurable Meta-Antennas**

*Daiju Ushikoshi, Riku Higashiura, Kaito Tachi, Ashif Aminulloh Fathnan, Suhair Mahmood, Hiroki Takeshita, Haruki Homma, Muhammad Rizwan Akram, Stefano Vellucci, Jiyeon Lee, Alessandro Toscano, Filiberto Bilotti, Christos Christopoulos and Hiroki Wakatsuchi*

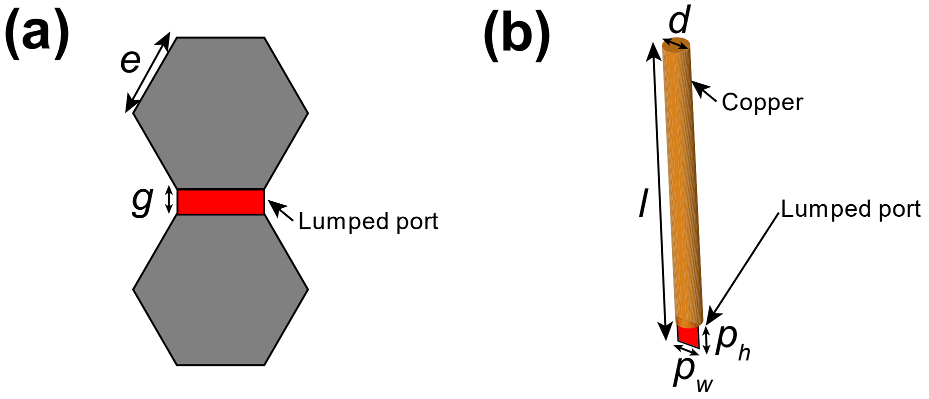


Figure S1: Design of the waveform-selective metasurface line and monopoles used in Fig. 1. (a) Patch design and (b) monopole. Their design parameters are given in Table S1. Additionally, the circuit values of Fig. 1b are shown in Table S2. The substrate was Rogers3010 (1.27 mm thick).

Table S1: Design parameters of the waveform-selective metasurface model shown in Figure S1.

| Parameter | Length [mm] |
|---|---|
| $e$ | 12 |
| $g$ | 1 |
| $l$ | 30 |
| $d$ | 1 |
| $p_h$ | 1 |
| $p_w$ | 1 |

Table S2: Circuit parameters of the waveform-selective metasurface model used in Fig. 1a to Fig. 1f.

| Parameter | Value (self-resonant frequency) |
|---|---|
| $C$ | 1 nF (200 MHz) |
| $R_C$ | 100 kΩ |
| $L$ | 100 µH (10 MHz) |
| $R_L$ | 5.5 Ω |
| $C_P$ | 100 pH (750 MHz) |
| $R_{CP}$ | 100 kΩ |
| $L_P$ | 1 mH (2.4 MHz) |
| $R_{LP}$ | 24 Ω |

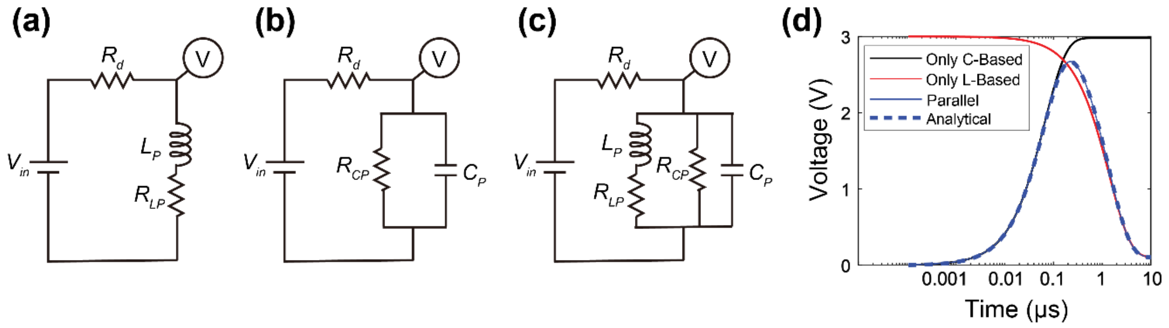


Figure S2: Schematics of DC circuit simulations where transient solver was used for (a) L-based, (b) C-based and (c) parallel type circuits. Here the equivalent diode resistance was represented by $R_d$ and set to $R_d$ = 680 Ω. The input voltage $V_{in}$ was 3V. Transient circuit components were identical to those used in the parallel waveform-selective metasurface presented in Table S4, i.e., $L_P$ = 1 mH, $R_{LP}$ = 24 Ω, $C_P$ = 100 pF, and $R_{CP}$ = 100 kΩ. (d) Simulated voltages over time for the C-based, L-based and parallel circuits using the aforementioned circuit parameters. The dashed line is the analytical result obtained from Eq. 10 of Ref [49].

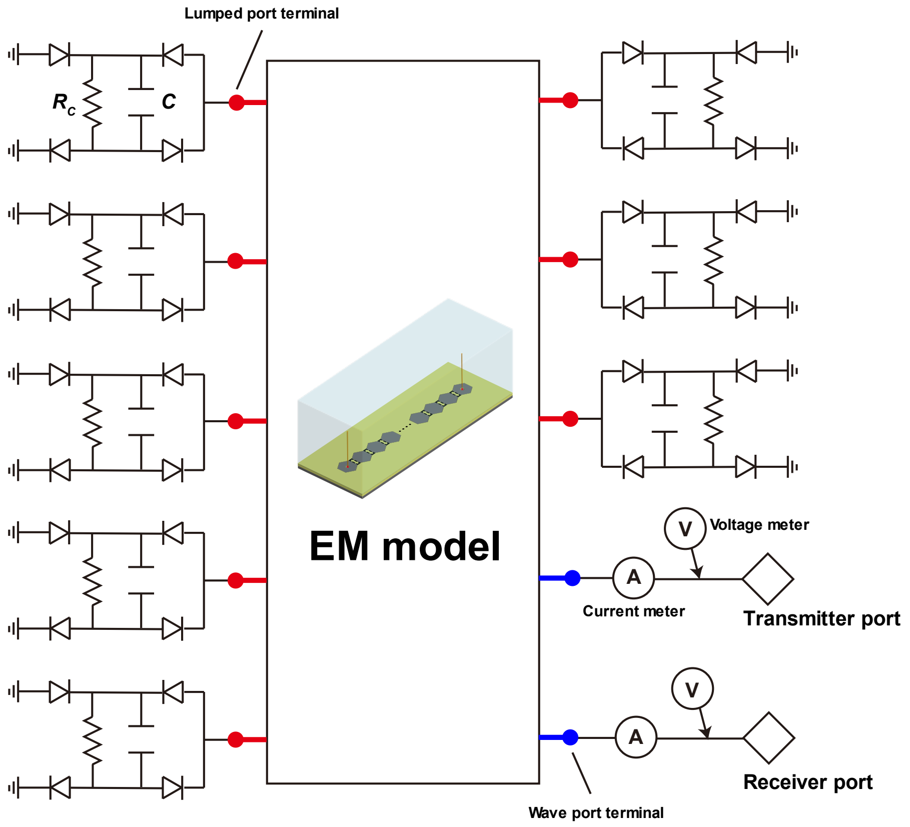


Figure S3: Schematic representing the co-simulation method in Fig. 1. An EM model was used in a circuit simulator and connected to lumped components and a signal source. Transmitter and receiver ports were used as the Tx and Rx. $R_C$s and $C$s were used for the C-based waveform-selective metasurface line, while other components were alternatively used for the L-based and parallel waveform-selective metasurface lines. More detail is provided under "Simulation Method" in the Methods Section.

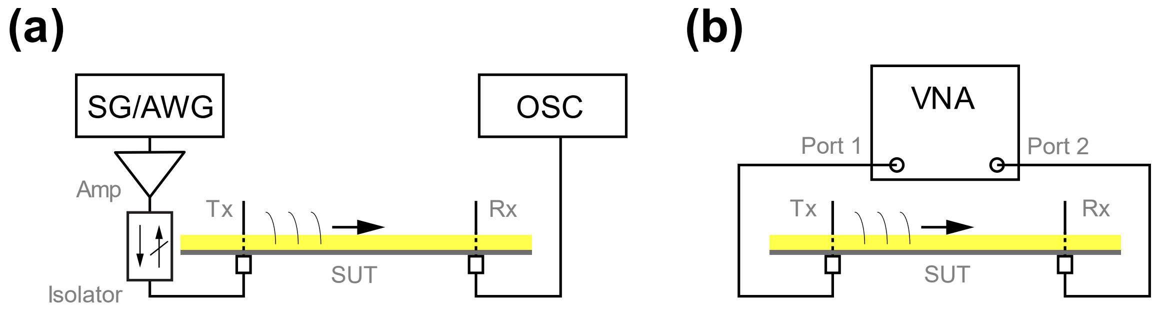


Figure S4: Setups for measuring (a) time-domain characteristics and (b) frequency-domain characteristics of Fig. 1 and Figure S8.

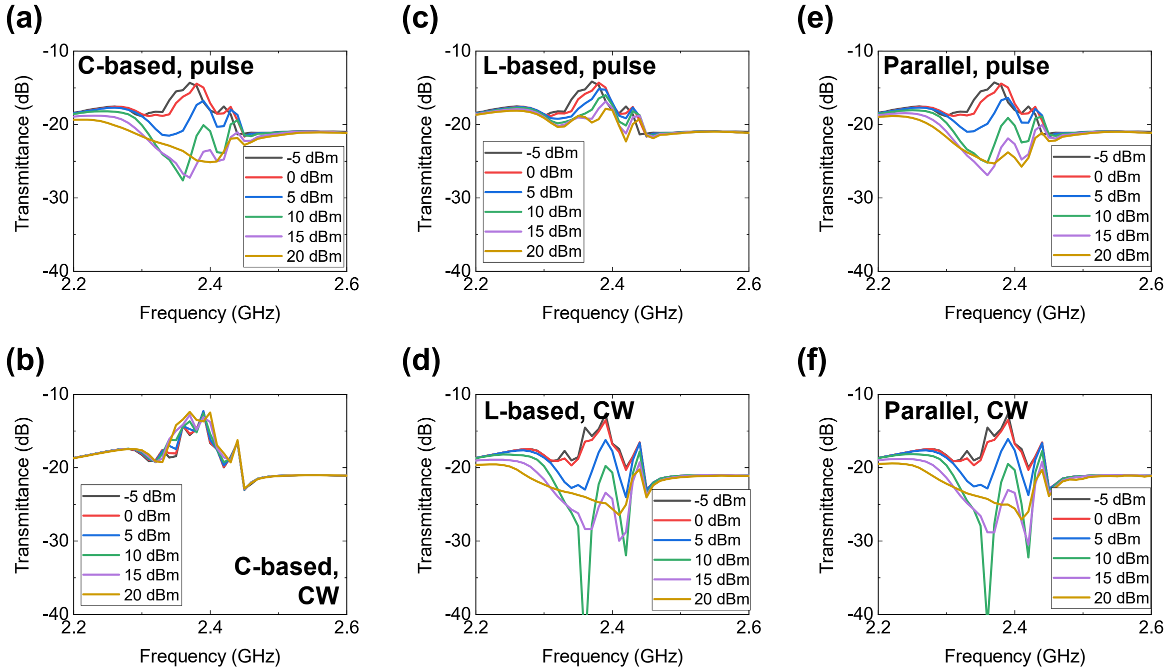


Figure S5: Simulation results of Fig. 1e with various power levels. Transmittances with (a, b) C-based, (c, d) L-based and (e, f) parallel waveform-selective metasurface lines. The top panels show the transmittances for 50-ns short pulses, while the bottom panels represent those for CWs. These results show that almost identical transmittances were obtained with low-power signals (e.g., -5 dBm) despite the differences in the input waveforms and the waveform-selective metasurfaces. This was because the power intensity was not large enough to turn on the diodes used. However, the transmittances differed from each other with sufficiently large power (e.g., 10 dBm).

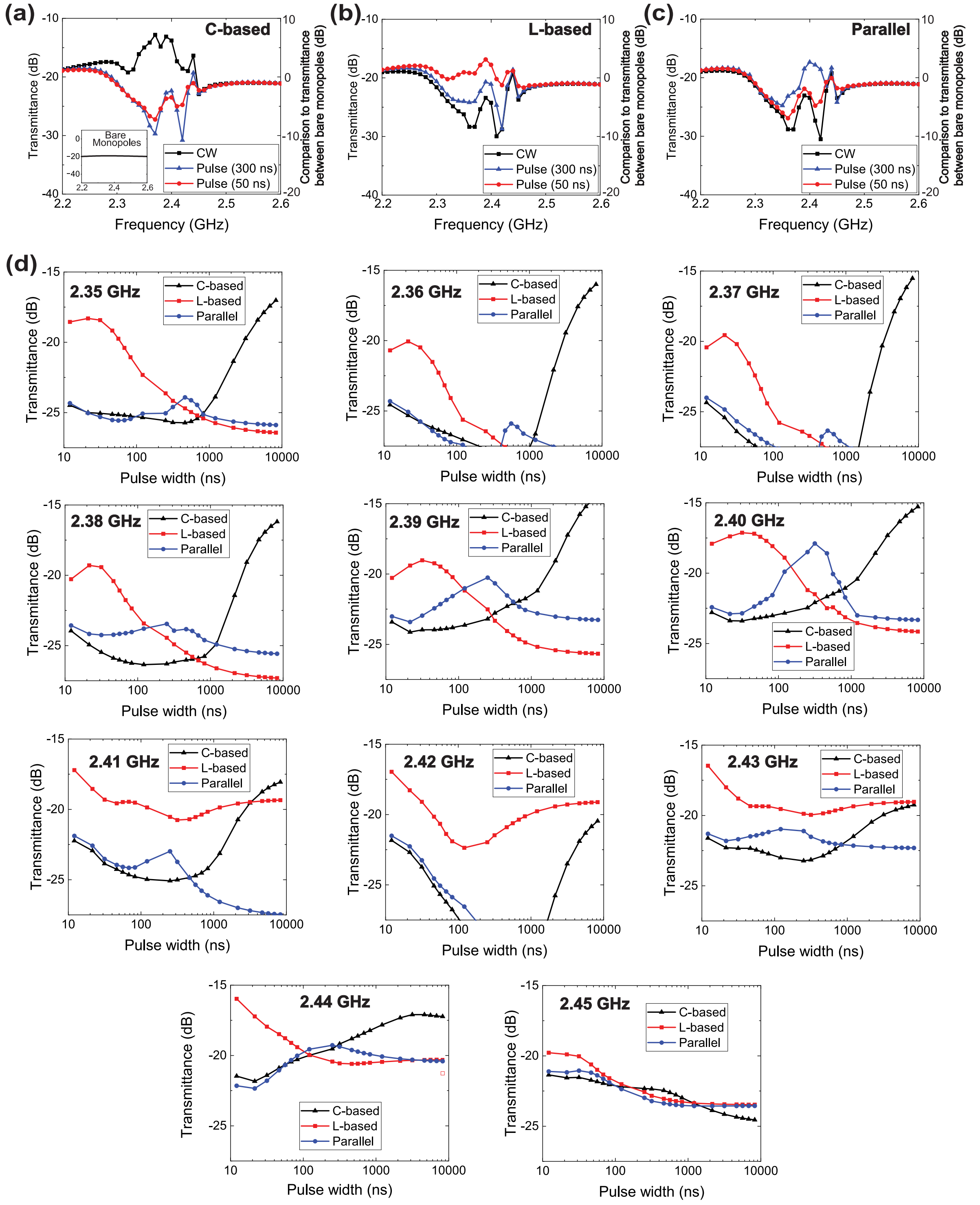


Figure S6: Simulation results of Fig. 1e with variation in pulse width. Transmittance over frequency using CWs, 300-ns pulses and 50-ns pulses for (a) C-based, (b) L-based and (c) parallel waveform-selective metasurfaces. In the right axes, the transmittance was compared to the one between two bare monopoles without the metasurfaces, showing an estimate for the transmission efficiency of each metasurface. We assumed that the transmittance between the bare monopoles was -20 dB according to the inset figure of (a), where the simulated transmittance between the bare monopoles was -19.61 ± 0.41 dB. (d) Transmittance of C-based, L-based and parallel waveform-selective metasurfaces with respect to pulse width variation near optimized frequency of 2.4 GHz.

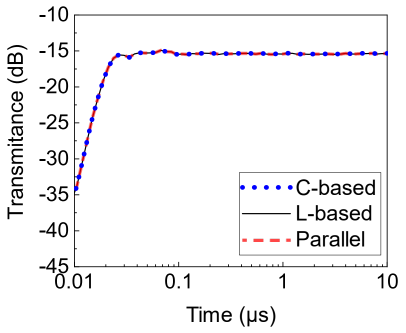


Figure S7: Simulation result of the left panel of Fig. 1f with -5 dBm. The signal generated from Tx took less than 100 ns to reach Rx for all three cases. Additionally, the three transmittances became constant and identical to each other.

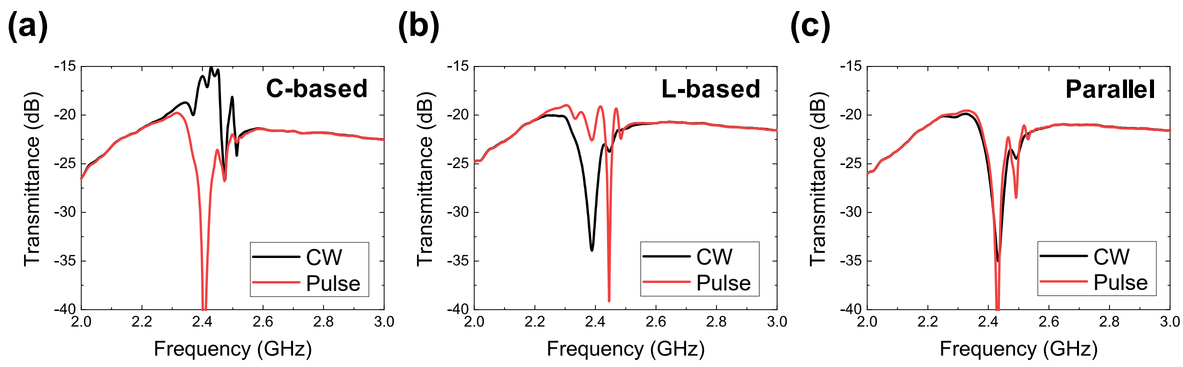


Figure S8: Measurement results of Fig. 1f in the frequency domain with 10 dBm. The results using (a) the C-based, (b) L-based and (c) parallel waveform-selective metasurface. In (a) transmittance for a CW was relatively larger than that for a 50-ns short pulse near 2.4 GHz due to the transient absorbing mechanism of the C-based waveform-selective metasurface. In contrast, the L-based waveform-selective metasurface more strongly absorbed a short pulse than a CW at the same frequency region, which appeared as a smaller transmittance for the CW in (b). Since both types of the circuit configurations were used in (c), the parallel waveform-selective metasurface reduced transmittances for a CW and a short pulse near 2.4 GHz.

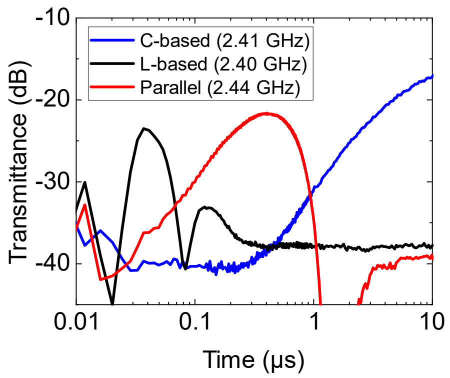


Figure S9: Transmittances of the measurement samples shown in the right panel of Fig. 1f with more optimized frequencies. The input power was fixed at 10 dBm. Results for other input powers and frequencies are seen in Figure S10 and Figure S11.

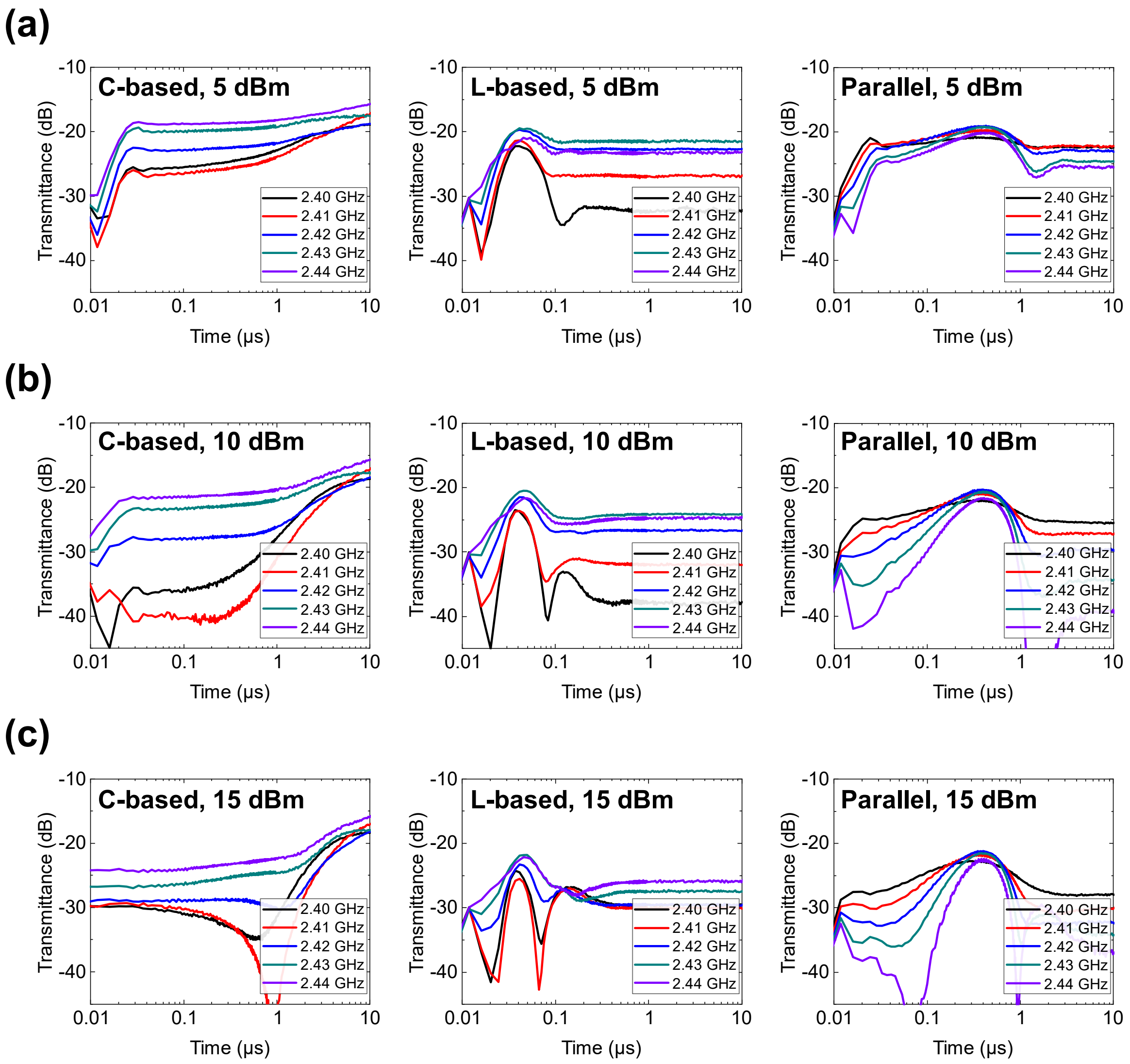


Figure S10: Transmittances of the measurement samples used in the right panel of Fig. 1f with different frequencies. The results using (a) 5 dBm, (b) 10 dBm and (c) 15 dBm. From the left panels to the right panels, the results correspond to the transmittances of the C-based, L-based and parallel waveform-selective metasurfaces, respectively. These results indicate that the transmittances tended to most effectively vary when the input power was set to 10 dBm or 15 dBm. At 2.40 GHz and 2.41 GHz, for instance, when the input power was 5 dBm, the transmittance of the C-based waveform-selective metasurface was approximately -25 dBm near 50 ns and 500 ns (see the black and the red curves of the left panel of (a)). However, by increasing the input power to 10 or 15 dBm, the transmittance became lower than -30 dBm. Note that near 50 ns and 500 ns, the L-based and the parallel waveform-selective metasurfaces increased the transmittances in the centre and the right panels of (a), which means that with 10 dBm and 15 dBm the difference between the three structures was maximized. In Figure S11, these results are plotted in accordance with frequency and input power.

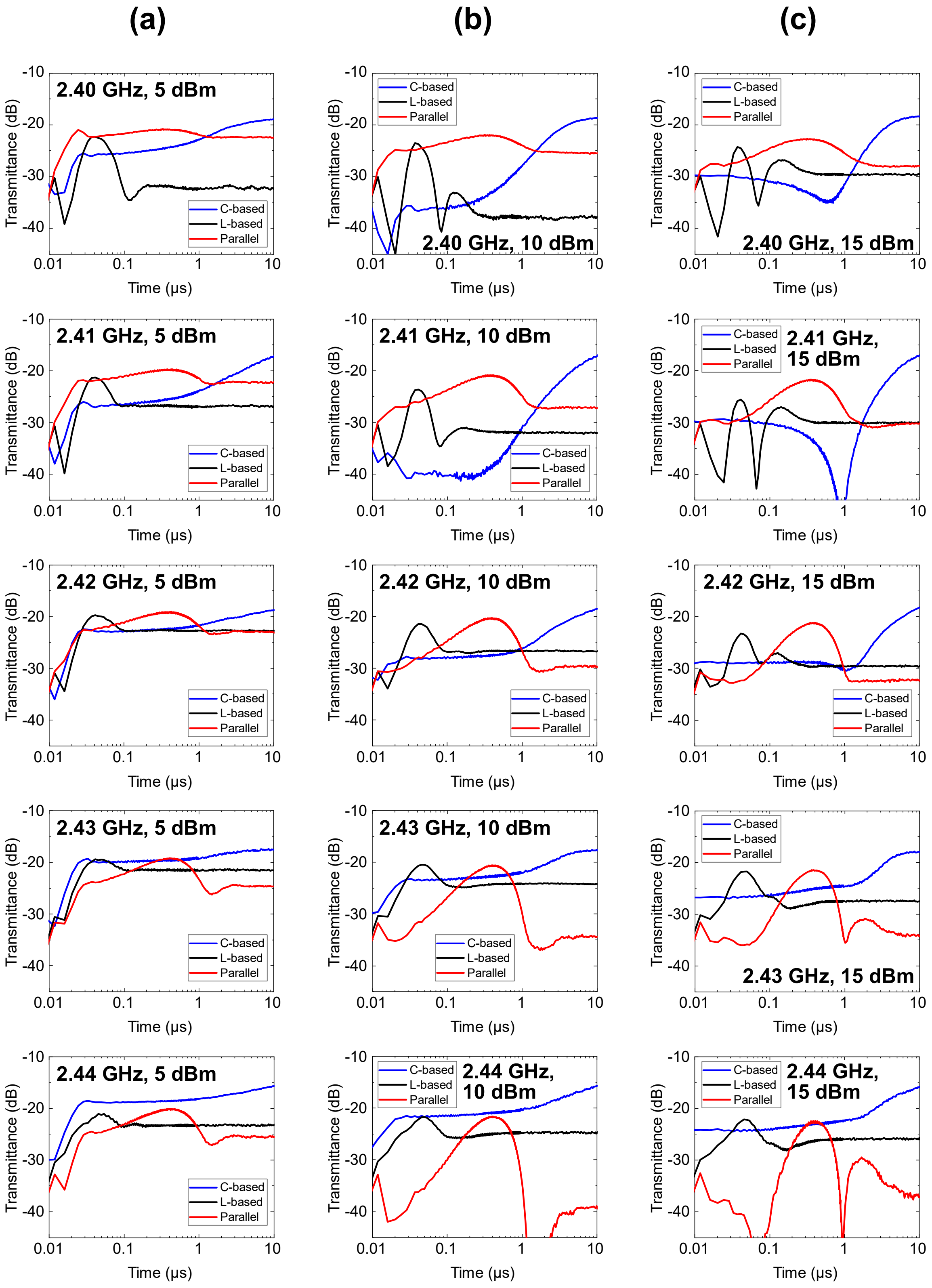


Figure S11: Transmittances of the measurement samples used in the right panel of Fig. 1f with different waveform-selective metasurfaces. The results using (a) 5 dBm, (b) 10 dBm and (c) 15 dBm. From the top panels to the bottom panels, the results correspond to the transmittances at 2.40, 2.41, 2.42, 2.43 and 2.44 GHz, respectively. In Figure S10, these results are plotted in accordance with input power and type of waveform-selective metasurface.

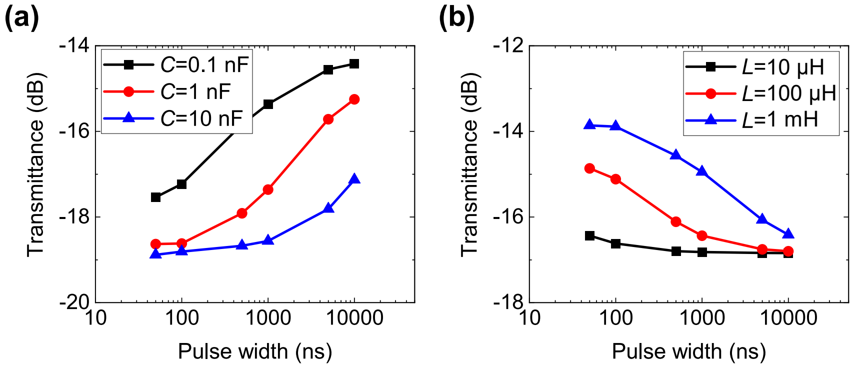


Figure S12: Simulated transmittances of (a) the C-based and (b) the L-based waveform-selective metasurface lines used in the left panel of Fig. 1f with various circuit values. By increasing $C$ and $L$, the transient responses were shifted to a large time range.

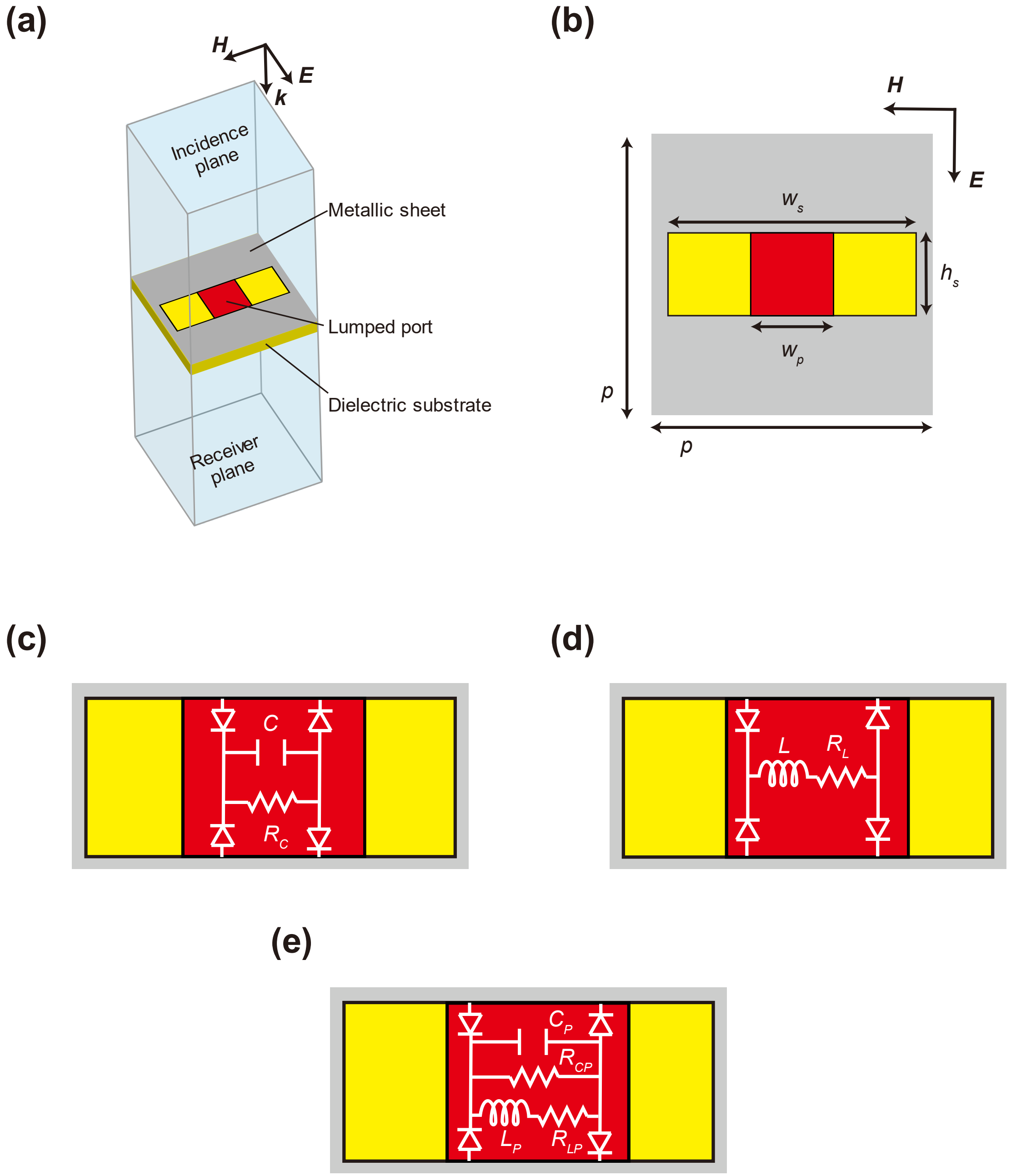


Figure S13: Single unit cell of the waveform-selective transmitting metasurface (slit structure) used for Fig. 3. (a) Periodic unit cell. Periodic boundaries were applied to the incident *E* and *H* planes. (b) The front surface of the periodic unit cell. (c, d and e) Circuit configurations of C-based, L-based and parallel waveform-selective transmitting metasurfaces. The design parameters are shown in Table S3 and Table S4. The substrate was Rogers3010 (1.27 mm thick).

Table S3: Design parameters of the waveform-selective metasurface model used in Figure S13b.

| Parameter | Length [mm] |
|---|---|
| $p$ | 17 |
| $w_s$ | 15 |
| $h_s$ | 5 |
| $w_p$ | 5 |

Table S4: Circuit parameters of the waveform-selective metasurface model used in Figure S13c to Figure S13e.

| Parameter | Value (self-resonant frequency) |
|---|---|
| $C$ | 1 nF (200 MHz) |
| $R_C$ | 10 kΩ |
| $L$ | 100 μH (10 MHz) |
| $R_L$ | 5.5 Ω |
| $C_P$ | 100 pF (750 MHz) |
| $R_{CP}$ | 100 kΩ |
| $L_P$ | 1 mH (2.4 MHz) |
| $R_{LP}$ | 24 Ω |

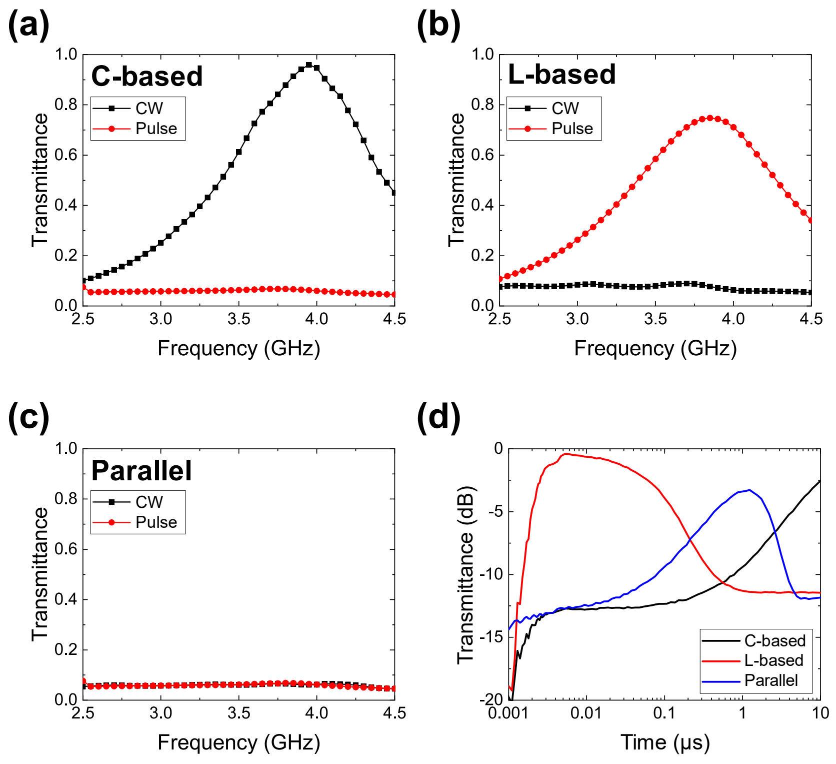


Figure S14: Simulated transmittances of the (a) C-based, (b) L-based and (c) parallel waveform-selective transmitting metasurfaces in Figure S13. The C-based waveform-selective metasurface more strongly transmitted CWs at approximately 4.0 GHz than short pulses, as the structure permitted induced electric charges to enter the diode bridge during an initial time period, which lowered the intensity of the intrinsic transmitting resonant mechanism of the slit structure. In contrast, the L-based waveform-selective metasurface showed more enhanced transmittance for short pulses than that for CWs. This was because at a steady state, the electromotive force of the inductor almost disappeared, which shortened the gap in the slit and weakened the intrinsic resonant mechanism of the structure. In the case of the parallel waveform-selective metasurface, both types of waveforms were poorly transmitted due to the presence of both types of circuit configurations. (d) Transient transmittances at 3.85 GHz. The incident power was set to 10 dBm. Due to the three different transient characteristics, the transmittances were maximized in different time slots.

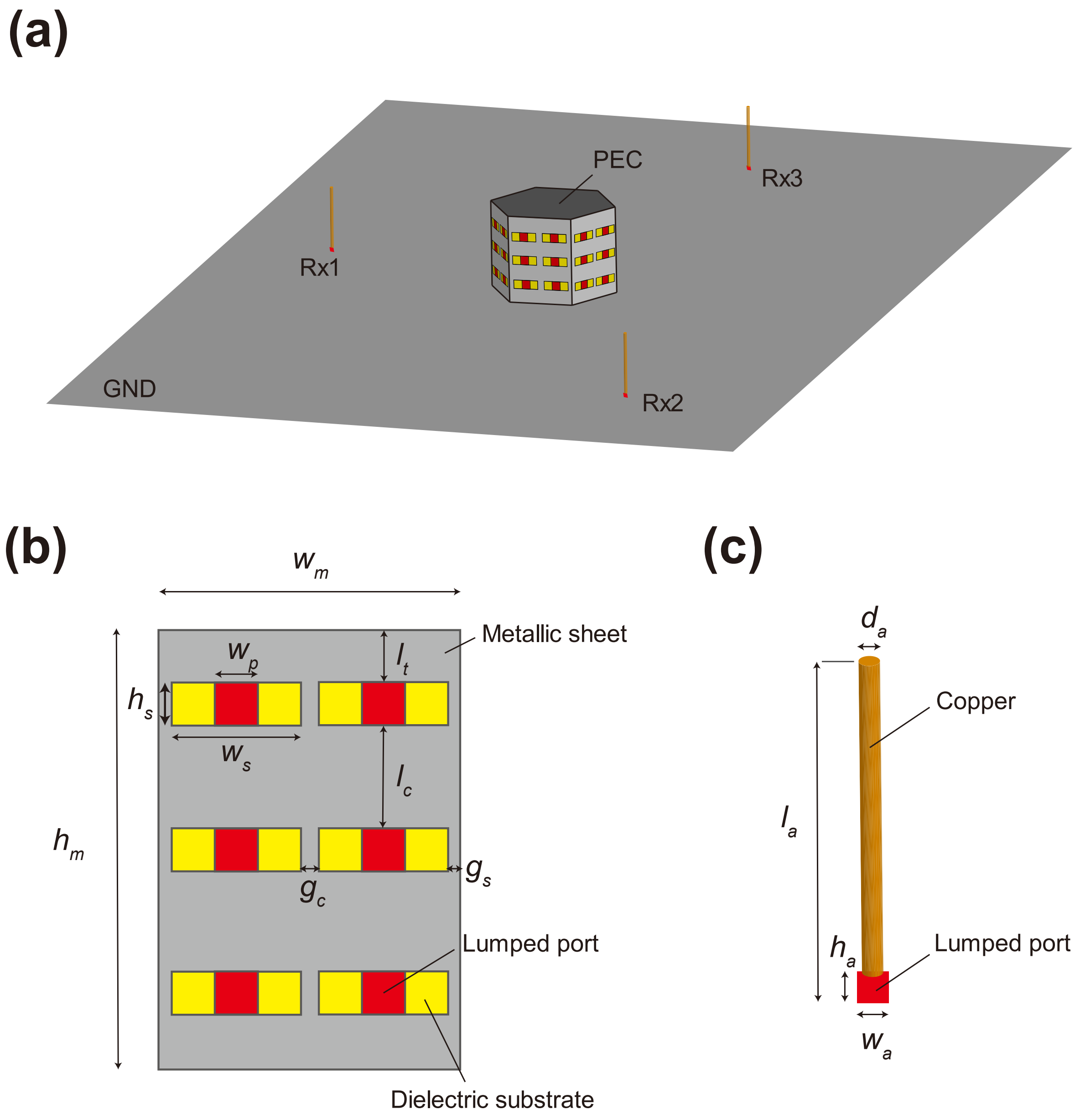


Figure S15: Antenna simulation model used for Fig. 3. (a) Entire model, (b) waveform-selective metasurface panel and (c) monopole. Dimensions and circuit values are given in Table S5 and Table S6. The substrate was Rogers3010 (1.27 mm thick). Rx1, Rx2 and Rx3 were respectively placed in front of L-based, parallel and C-based waveform-selective metasurface panels.

Table S5: Design parameters of the waveform-selective metasurface model used in Figure S15 (i.e., in Fig. 3).

| Parameter | Length [mm] |
|---|---|
| $w_m$ | 35 |
| $h_m$ | 51 |
| $w_s$ | 15 |
| $h_s$ | 5 |
| $w_p$ | 5 |
| $l_t$ | 6 |
| $l_c$ | 12 |
| $g_c$ | 2 |
| $g_s$ | 1.5 |
| $l_a$ | 18 |
| $d_a$ | 1 |
| $w_a$ | 1 |
| $h_a$ | 1 |

Table S6: Circuit parameters of the waveform-selective metasurface model used in Figure S15 (i.e., in Fig. 3).

| Parameter | Value (self-resonant frequency) |
|---|---|
| $C$ | 10 nF (2 GHz) |
| $R_C$ | 100 kΩ |
| $L$ | 100 μH (10 MHz) |
| $R_L$ | 5.5 Ω |
| $C_P$ | 1 nF (200 MHz) |
| $R_{CP}$ | 100 kΩ |
| $L_P$ | 1 mH (2.4 MHz) |
| $R_{LP}$ | 24 Ω |

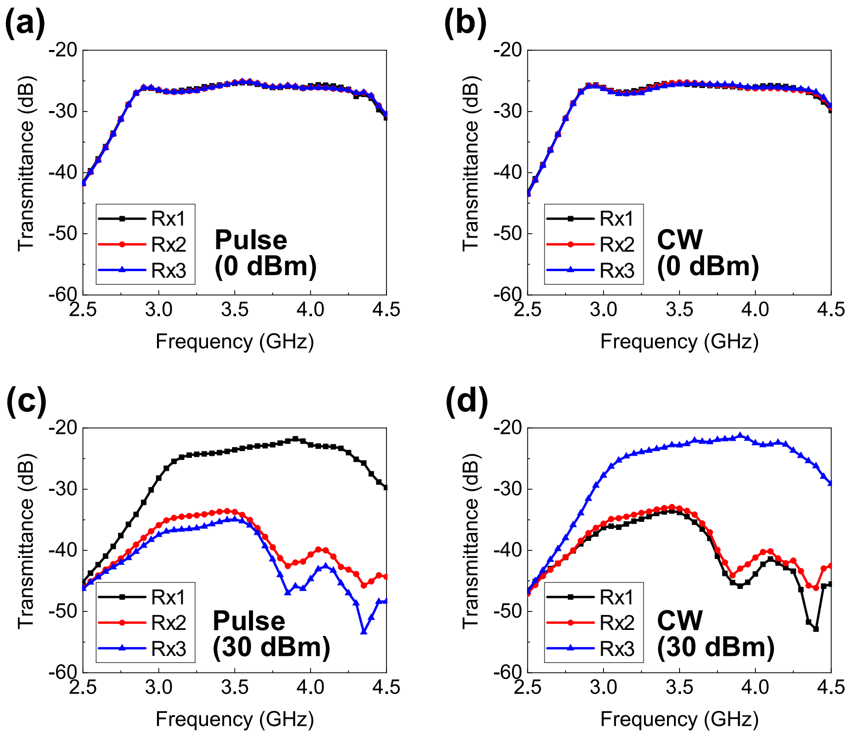


Figure S16: Simulated frequency dependence of the antenna shown in Fig. 3, composed of 3 different types of waveform-selective metasurfaces. (a) 0-dBm short pulses. (b) 0-dBm CWs. (c) 30-dBm short pulses. (d) 30-dBm CWs. As shown in (a) and (b), there was almost no difference between the transmittances for short pulses and those for CWs because the diodes used were not turned on. In contrast, (c) and (d) showed clear differences. Specifically, the transmittance of Rx1 was maintained at a large level for the 30-dBm short pulses between 3.2 and 4.5 GHz in (c), as the L-based waveform-selective metasurface deployed in front of Rx1 strongly transmitted the signal generated from the transmitter compared to the C-based and parallel waveform-selective metasurfaces. In the case of the 30 dBm CWs, the C-based waveform-selective metasurface effectively transmitted signals to Rx3, which appeared as a relatively large transmittance in (d). The parallel waveform-selective metasurface poorly transmitted both the 30-dBm pulses and the 30-dBm CWs due to the presence of both circuits, appearing as limited transmittances in (c) and (d).

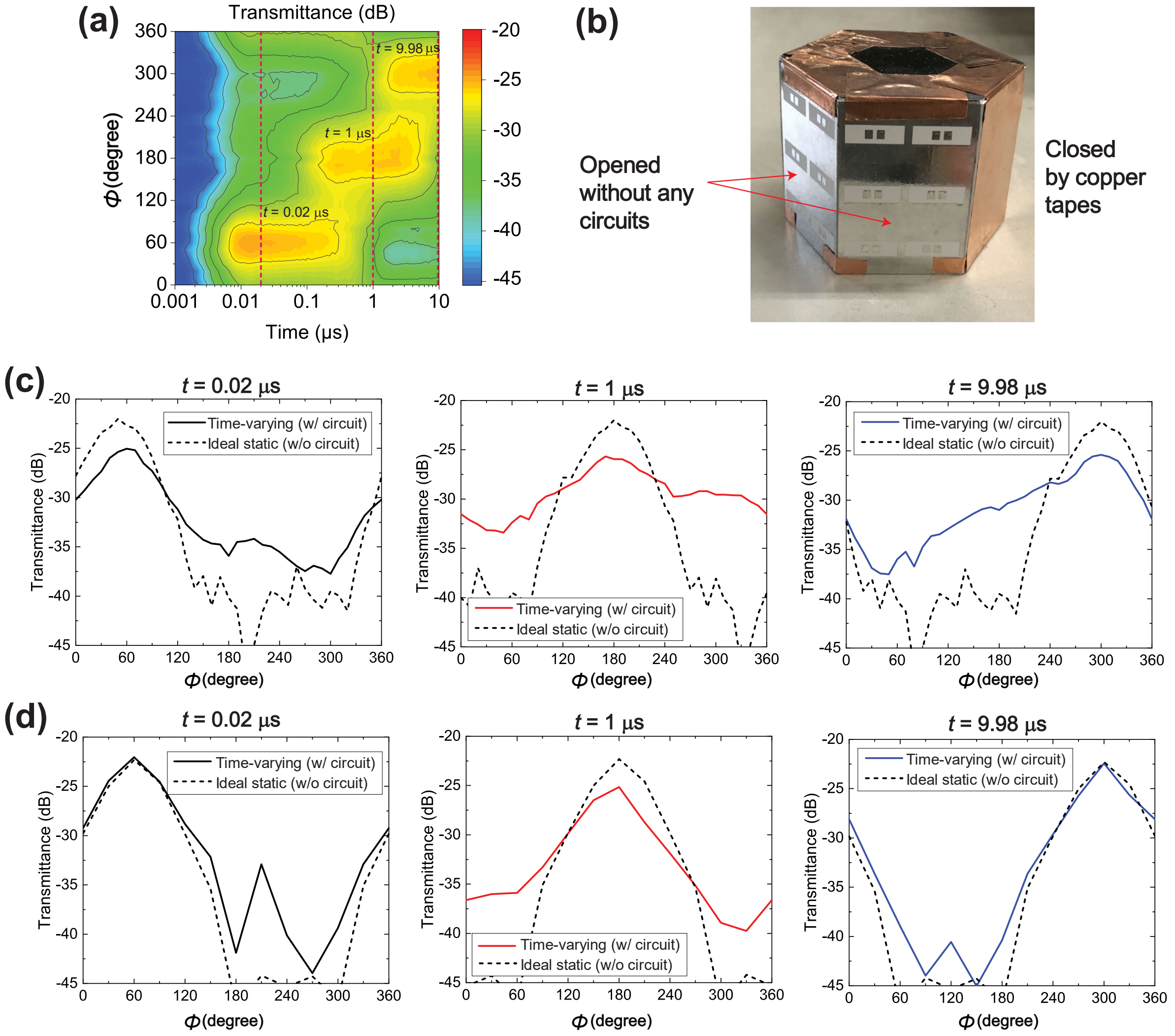


Figure S17. (a) Measured time-varying transmittance from the metasurface-based antenna shown in Fig. 3g with various far-field angles $\phi$. Three vertical dashed lines indicate time samples where the far-field patterns were presented in Fig. 3g (also presented here in Figure S17c). (b) Metasurface sample in ideal directive condition. Slits in two hexagonal sides were opened without circuits, while the others were fully closed by copper tapes. No time variation was observed in this ideal directive condition (static). (c) Comparison between the ideal and realized far-field patterns from the measurement results. In the right axis of the right panel of Fig. 3g, the measured far-field patterns were subtracted by the maximum level of the ideal directive condition, which gave a difference of around -5 dB. (d) Comparison between the ideal and realized far-field patterns from the simulation results. Here, a better agreement was obtained between the ideal and realized field power than those obtained in the measurement. This was presumably because additional losses introduced during the fabrication process reduced the measured transmittance.

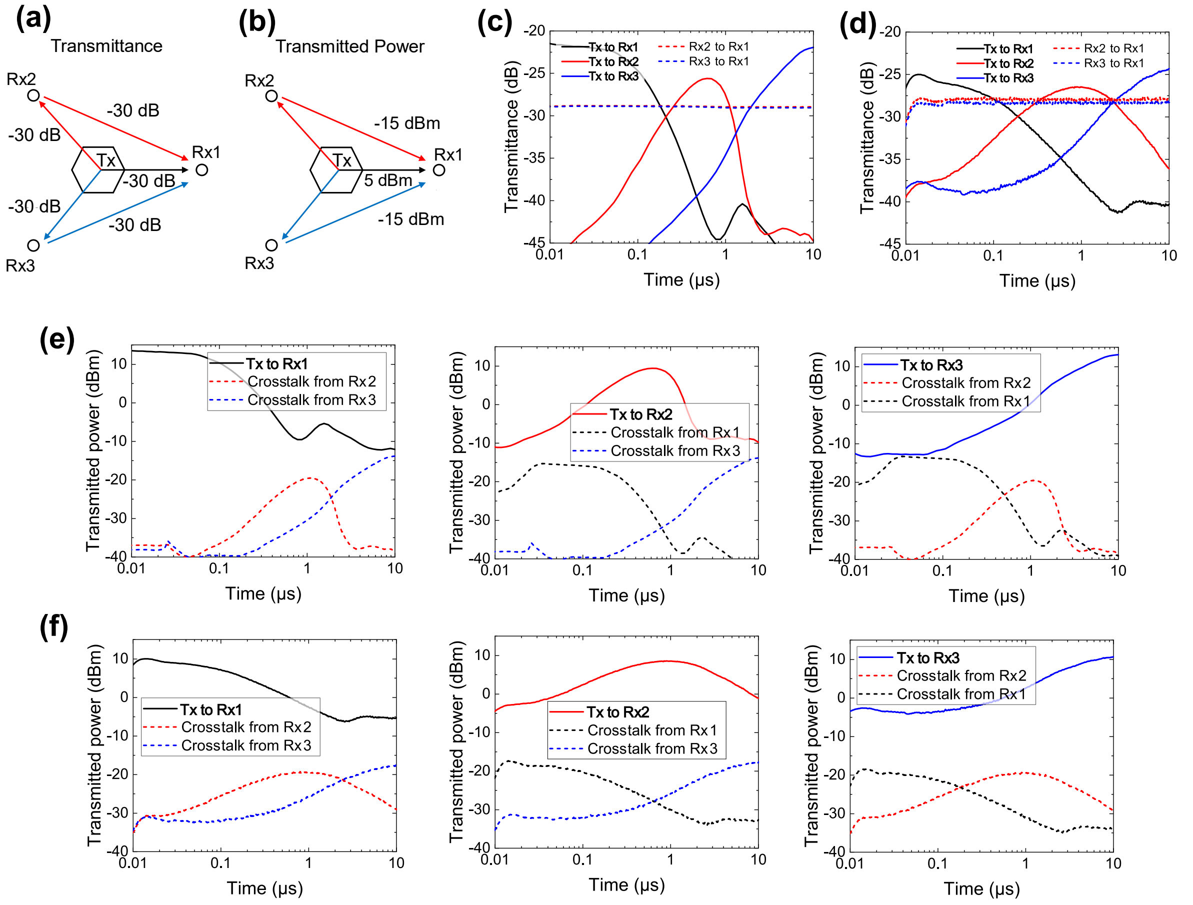


Figure S18: Characterization of crosstalk between external antennas with the proposed metasurface. (a) Schematic of interference paths. Rx1 also received secondary reflection from both Rx2 and Rx3. The estimated power in each path is indicated. (b) Transmitted power calculated using the transmittance in (a) when the source power was 35 dBm. While the main path from Tx to Rx1 gave 5 dBm power, the crosstalk from Rx2 and Rx3 was only -15 dBm. (c-d) Transmittance over time for all antennas outside the hexagonal metasurface prism in (c) simulation and (d) measurement. (e-f) Power received through the main path, i.e., corner directions of each metasurface panel, compared to the crosstalk both in (e) simulation and (f) measurement.

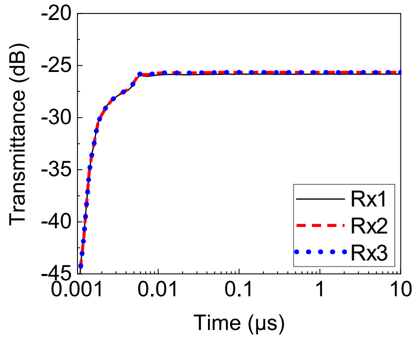


Figure S19: Simulated transient transmittance of the antenna shown in Fig. 3 and composed of 3 different types of waveform-selective metasurfaces at 3.85 GHz with 0 dBm. The simulation result shows that all the receivers observed the signal in almost the same manner as the diodes used were not turned on. This result also indicates that the signal took approximately 10 ns to reach the receivers. From 10 ns to 10 μs, the transmittance was fixed at a constant value since the transmitter behaved as an ordinary omnidirectional antenna. The related frequency-domain profiles are shown in Figure S16.

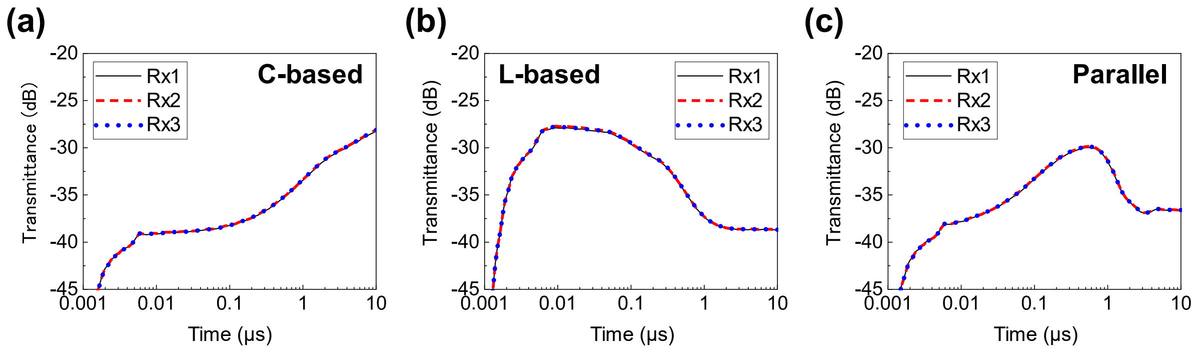


Figure S20: Simulated transient transmittance of the antenna shown in Fig. 3 but composed of only one of the three waveform-selective metasurfaces at 3.85 GHz with 30 dBm. The results using (a) C-based, (b) L-based and (c) parallel waveform-selective metasurfaces. Compared to Figure S18, although all the receivers received the signal in the same manner, the transmittance profile was shown to vary in the time domain.

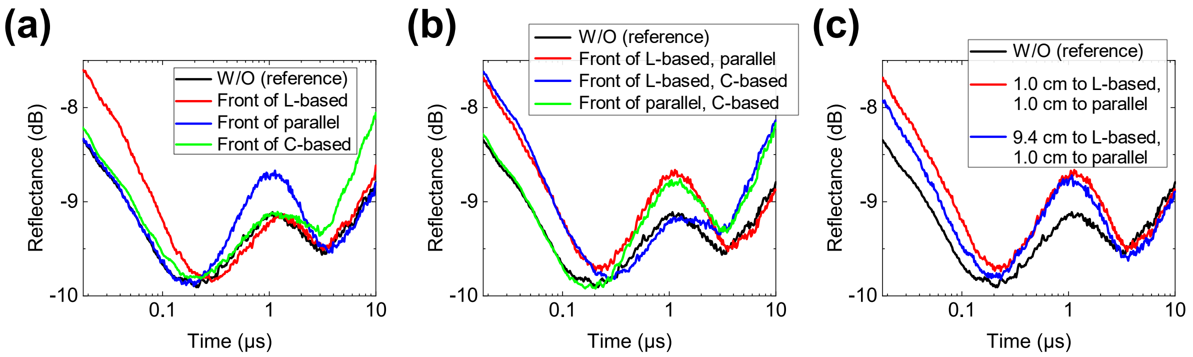


Figure S21: Measured reflectances of the passive variable sensor shown in Fig. 4. (a) Measurement results using only one copper plate. Measurement results using two copper plates with (b) the same distances and (c) different distances. Increases in reflectance of (a), (b) and (c) are seen in Fig. 4b, Fig. 4c and Fig. 4d, respectively.

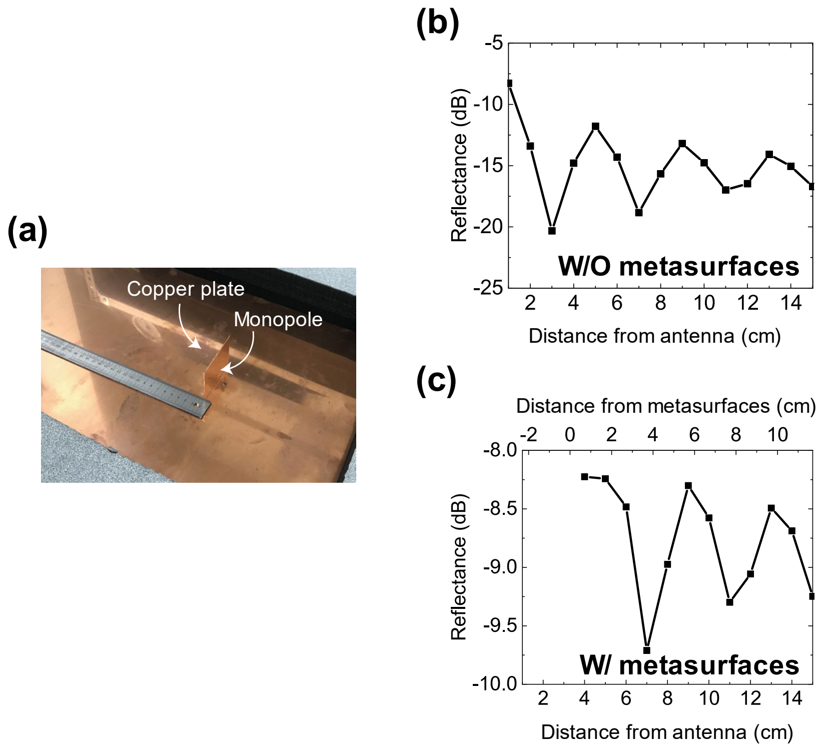


Figure S22: Measured reflectances of the passive variable sensor shown in Fig. 4 with various distances between the transmitting monopole and copper plate. (a) Image of the measurement without waveform-selective metasurface panels. Results (b) without and (c) with waveform-selective metasurface panels. In (c) the copper plate was positioned in front of the L-based waveform-selective metasurface panels. The result of (c) was obtained around 50 ns. These results indicate that there appeared a standing wave between the monopole and the copper plate, which affects the sensing performance. However, this influence can be mitigated by improving the impedance matching of the monopole. Nonetheless, these results show that the envelop curves in (b) and (c) gradually decreased by increasing the distance from the monopole antenna. Thus, if the impedance matching issue is addressed then the sensing performance is expected to be more enhanced to detect the distance from a scattering object.

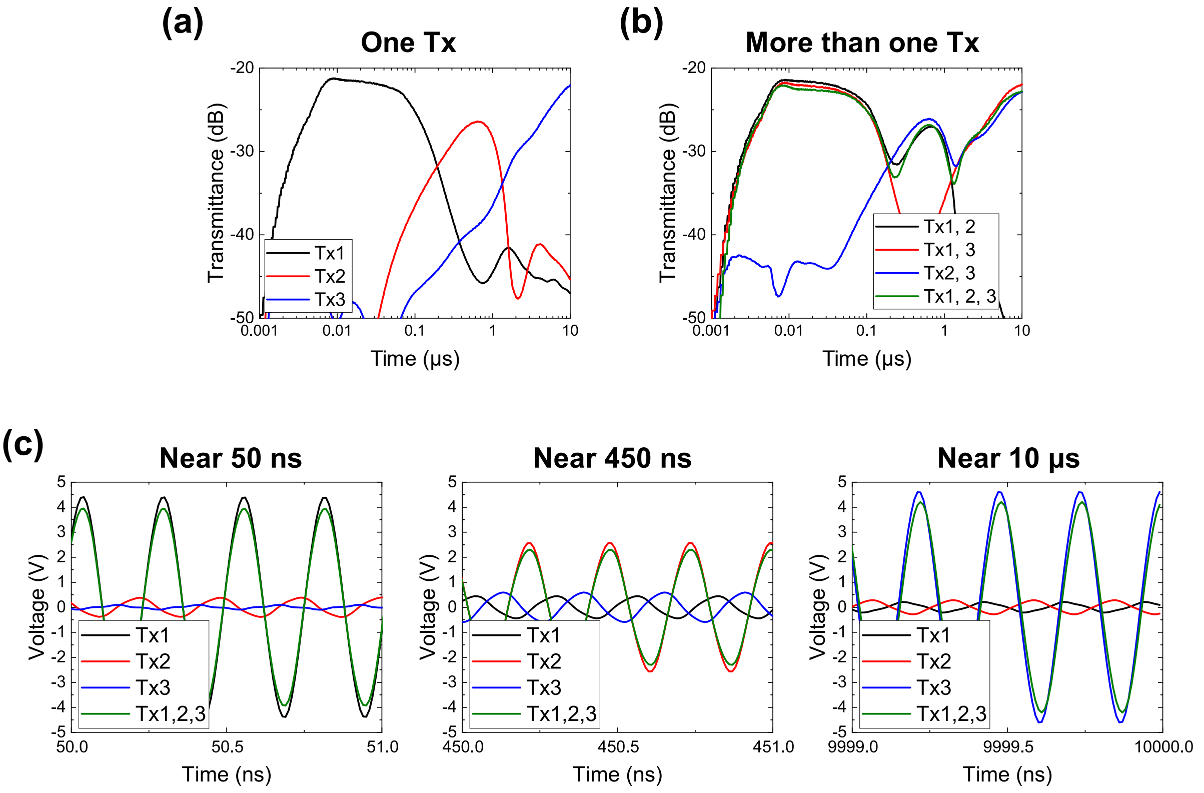


Figure S23: Additional simulation results for Fig. 5 with the distance between the transmitters and the receiver increased to 200 mm (representing a more realistic far-field distance). The input power was increased to 45 dBm to turn on the diodes used. (a) Transmittances with a single source. (b) Transmittances with more than one source. (c) Voltages in the time domain. Almost the same conclusion was drawn as that of Fig. 5 except minor discrepancies including different magnitudes in transmittances. More importantly, however, this figure also establishes that waveform-selective reception is achievable even under simultaneous incidences and with far-field radiation.

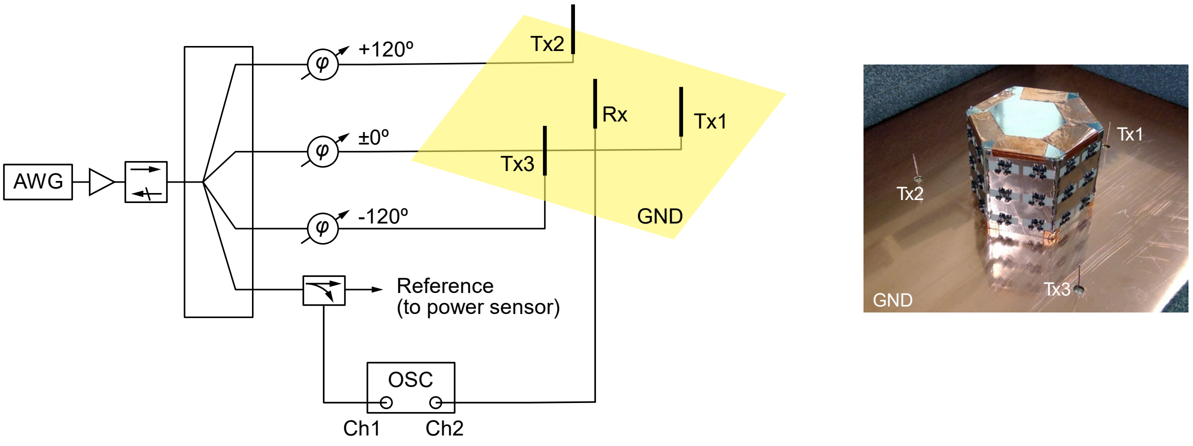


Figure S24: Measurement setup for the simultaneous incidences in Fig. 5. Tx1, Tx2 and Tx3 were respectively placed in front of L-based, parallel and C-based waveform-selective metasurface panels. A detailed description of the setup is presented under “Measurement Method” in the Methods Section.

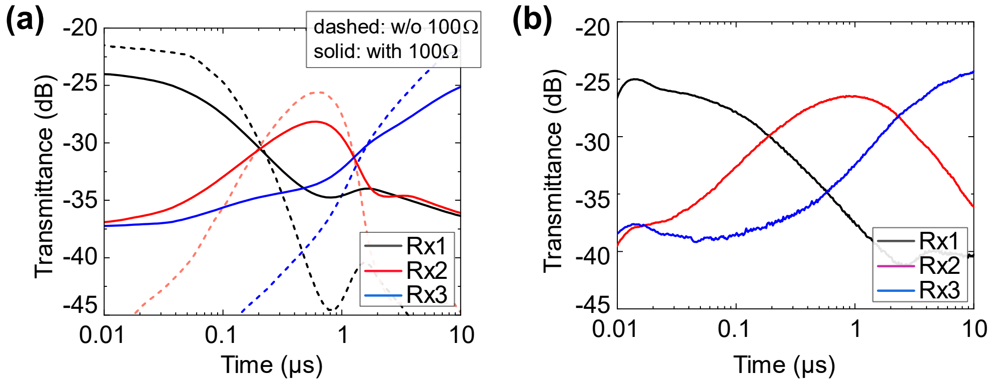


Figure S25. (a) Simulated transmittance of the metasurface antenna used in Fig. 3. The input frequency was set to 3.85 GHz with a 30-dBm power level. An additional resistor of 100 Ω was connected to each diode bridge in series (solid), compared to the original simulation result without the additional resistance (dashed). The resistance was deliberately added to resemble additional losses introduced during the fabrication process. (b) Measured transmittance using the same setup (also presented in Fig. 3c).

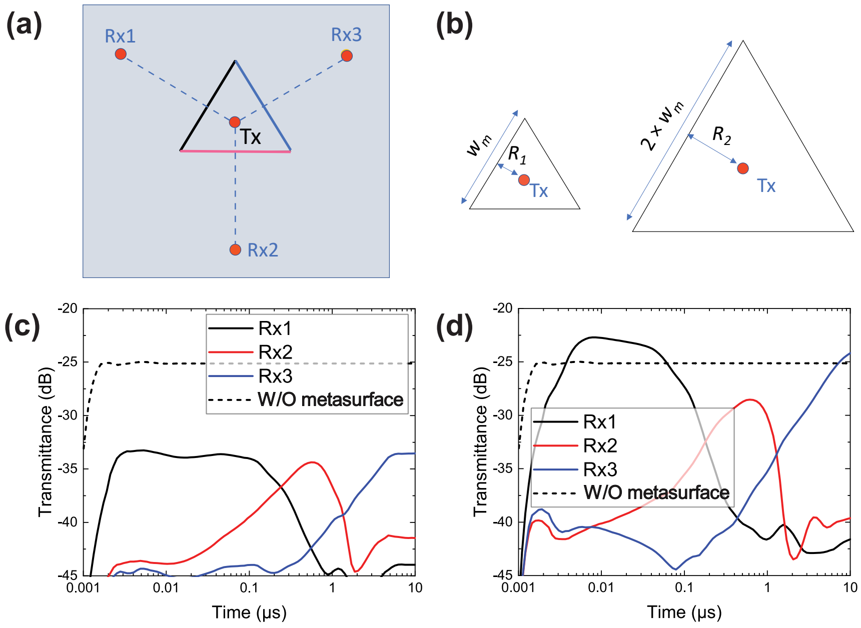


Figure S26: (a) Schematic of the equilateral triangle metasurface antenna simulation. (b) Comparison of two triangle metasurfaces having different inscribed radius of $R_1$ = 10.1 mm (~ 0.13$\lambda$) and $R_2$ = 20.1 mm (~ 0.26$\lambda$). In both cases the distance between the transmitter (Tx) and the receivers (Rx1-Rx3) was 200 mm. Transmittance of the metasurface antenna over time for the metasurface with (c) the small triangle shape and (d) the large triangle shape. The transmittance was significantly reduced in the small triangle case compared to the large triangle case due to the effect of coupling as well as standing wave that were amplified by the smaller distance between the monopole antenna and the metasurface panels (equivalent to the inscribed radius $R$).
